# Cancer prevention and interception studies registered in ClinicalTrials.gov: a landscape analysis of trial characteristics and reporting gaps

**DOI:** 10.3389/fepid.2026.1943268

**Published:** 2026-09-28

**Authors:** Martha Fors, Paloma González, Ariel Torres, Lizbeth Silva-Guayasamín, Christian Silva-Sarabia

**Affiliations:** 1 Escuela de Medicina, Facultad de Ciencias de la Salud, Universidad de Las Américas Redondel del Ciclista Sin Número, antigua Via A Nayón, Quito, Pichincha, Ecuador; 2 Biblioteca, Universidad de Las Américas, Quito, Ecuador; 3 Universidad Tecnológica Indoamérica, Quito, Ecuador; 4 Universidad Nacional del Chimborazo (UNACH), Riobamba, Ecuador

**Keywords:** cancer interception, cancer prevention, chemoprevention, clinical trial registries, ClinicalTrials.gov, premalignant lesions

## Abstract

**Background:**

Cancer interception has emerged as a prevention-oriented approach that seeks to intervene during early stages of carcinogenesis, before the development of clinically manifest invasive disease. However, the extent to which prevention and interception research is represented and classified in clinical trial registries remains unclear.

**Methods:**

We conducted a cross-sectional, registry-based analysis of cancer prevention and interception-related studies registered in ClinicalTrials.gov through September 2025. Records identified using terms related to cancer prevention, interception, chemoprevention, risk reduction, premalignant lesions, precursor lesions, and minimal residual disease were screened for eligibility. Study characteristics, including study type, recruitment status, participant eligibility, enrollment, intervention model, allocation, masking, cancer localization, study phase, and funding category, were summarized descriptively. Associations between categorical variables were assessed using exact tests when appropriate.

**Results:**

Among 209 eligible studies, 157 (75.1%) were interventional and 52 (24.9%) were observational. Most studies included adults and older adults, and 52.6% were classified as completed. Among interventional studies, randomized allocation and parallel-group designs predominated, while most studies were unmasked. Gynecological and gastrointestinal malignancies were the most frequently represented cancer localizations. Funding was classified as ‘Other’ in most records, reflecting a heterogeneous registry category that could not be further disaggregated. Study type was associated with funding category (*p* = 0.028), whereas the association between study type and cancer localization was not statistically significant (*p* = 0.09, with a Cramér's V of 0.32). Among interventional studies, 54 (34.4%) focused on early detection and diagnosis, aligning with the registry's ’Screening’ and ‘Diagnostic’ fields. Cancer interception and post-treatment disease control accounted for 52 studies (33.1%) but lacked distinct registry designations, while 51 (32.5%) addressed primary prevention and chemoprevention, corresponding to the ‘Prevention’ category.

**Conclusions:**

Cancer prevention and interception research registered in ClinicalTrials.gov is heterogeneous and encompasses distinct clinical objectives. Our findings highlight the need for explicit operational definitions and more granular, standardized reporting fields to improve the visibility, interpretability, and comparability of preventive oncology.

## Introduction

Oncology has traditionally focused on the diagnosis and treatment of clinically established cancer. Advances in molecular biology, genomics, imaging, and biomarker science have increasingly shifted attention toward earlier stages of carcinogenesis, when disease risk or biological progression may be detectable and potentially modifiable. This has contributed to growing interest in cancer prevention and interception strategies designed to reduce the probability of progression to clinically manifest invasive disease (1–4).

The concept of disease interception is not completely uniform across the literature. In general, interception refers to an intervention delivered during an early biological stage of disease development, often before the onset of clinically apparent symptoms, with the aim of preventing or delaying progression. In cancer, this may involve individuals with inherited or acquired risk, molecular abnormalities, premalignant lesions, precursor conditions, or other biologically defined states associated with a clinically meaningful risk of progression. The concept therefore overlaps with, but should not be considered synonymous with, primary prevention, screening, or early detection. Primary prevention aims to reduce the probability of cancer development before premalignant disease is established, whereas screening and early detection primarily seek to identify cancer or precursor lesions at an earlier stage. Interception, in contrast, focuses on modifying an existing biological, molecular, or premalignant state to prevent or delay progression to clinically manifest disease (5–7). A recent framework proposes three complementary categories of cancer prevention activities: cancer prophylaxis, which aims to prevent initiation of the carcinogenic process; cancer interception, which seeks to halt or reverse disease progression before clinically manifest cancer develops; and cancer mitigation, which focuses on early identification and management of cancer to reduce morbidity, mortality, and treatment-related harm (6).

To provide conceptual clarity along the prevention continuum, we explicitly distinguished three operational categories: (1) Primary Prevention, targeting individuals without detectable premalignant disease who are at increased risk of cancer; (2) Early Detection and Diagnosis, encompassing screening and diagnostic strategies intended to identify occult or early-stage malignant or precursor lesions; and (3) Cancer Interception and post-treatment Disease Control, encompassing interventions delivered during an early molecular, genetic, histopathological, or premalignant state to prevent or delay progression to invasive cancer, as well as interventions targeting minimal residual disease or recurrence following treatment of established cancer (8, 9).

The growing interest in precision prevention and cancer interception raises an important methodological question: how is this emerging research area represented in clinical trial registries? A registry-based assessment can provide information on the types of studies being conducted, the cancers and populations receiving research attention, the designs and phases used, and the extent to which registry fields adequately describe prevention-oriented research. ClinicalTrials.gov was selected as the primary data source because it is a large, publicly accessible registry that provides standardized information on study characteristics, eligibility, interventions, study design, recruitment status, and funding across a broad range of clinical studies. However, ClinicalTrials.gov does not encompass all clinical studies registered internationally, and findings based on this registry should therefore be interpreted as a representation of the registered landscape rather than a complete census of global clinical research. Despite increasing conceptual interest in cancer interception, the registered clinical research landscape has not been comprehensively characterized. It remains unclear how studies related to prevention, interception, premalignant disease, and minimal residual disease are distributed across cancer localizations, study designs, participant populations, and funding categories, and to what extent the registry-defined classifications correspond to these clinically distinct research objectives.

A major conceptual barrier in preventive oncology is the historical tendency to conflate risk reduction, early detection, and cancer interception under a single umbrella term. However, these paradigms target fundamentally distinct biological windows and clinical populations. While primary prevention focuses on healthy individuals to avoid initial carcinogenic exposure, and screening seeks to identify pre-existing occult malignancy or advanced lesions, cancer interception operates in an intermediate biological space delivering targeted pharmacological, immunological, or molecular interventions during pre-invasive, premalignant, or minimal residual states to actively halt progression toward invasive disease. Despite the growing recognition of precision interception supported by advancements in liquid biopsies and molecular profiling, clinical trial registries lack dedicated metadata structures to isolate these studies, frequently scattering them across miscellaneous or therapeutic categories. This structural ambiguity obscures the true landscape of preventive research and hinders evidence synthesis (10–12).

Therefore, this study aimed to characterize cancer prevention and interception-related studies registered in ClinicalTrials.gov through September 2025, with emphasis on study design, cancer localization, participant eligibility, recruitment status, study phase, intervention characteristics, and funding. A secondary aim was to assess the extent to which the registry-defined classifications and available study information characterize the heterogeneous research landscape surrounding cancer prevention and interception.

## Methods

### Study design and data source

We conducted a cross-sectional, registry-based study using data from ClinicalTrials.gov, a publicly accessible registry maintained by the U.S. National Library of Medicine. The analysis was based on study-level registry information available through September 2025. The individual registered study was the unit of analysis.

### Search strategy and study identification

We conducted a comprehensive search on ClinicalTrials.gov (https://clinicaltrials.gov/) on September 30, 2025, using the following registry fields: ‘Condition or disease’ and ‘Other terms.’ The search syntax utilized the Boolean operator ‘OR’ to combine the following terms: *cancer*, *oncology*, *interception*, *precision prevention*, *chemoprevention*, *early intervention*, *risk reduction*, *minimal residual disease*, *premalignant disease*, *precancer*, *precursor lesions*, *carcinoma in situ*, *intraepithelial neoplasia*, *dysplasia*, and *adenoma*. We applied filters for ’Study Type’ (Interventional and Observational) and included all recruitment statuses (Active_not_recruiting, Enrolling_by_invitation, Suspended, Unknown and Withdrawn). The search terms were combined using the Boolean operator ‘OR’ to maximize the identification of studies related to the cancer prevention and interception continuum. The search was conducted without restrictions regarding cancer site, participant age, sex, study phase, intervention type, or geographic location. Filters were applied to include both interventional and observational studies, and all available recruitment statuses were considered. Data were exported as a standardized Comma-Separated Values (CSV) file, ensuring that the full registry record was retrieved for each study. This search strategy ensures full transparency and reproducibility of the data collection process.

The initial search identified 296 records. After screening, 87 records were excluded because they were unrelated to the cancer prevention or interception research question (*n* = 75) or had insufficient information for the intended classification (*n* = 12), resulting in 209 eligible studies.

To minimize misclassification bias during the initial screening of the 296 retrieved records, terms that frequently overlapped across domains (such as ‘chemoprevention’ combined with ‘early detection’) underwent a dual-reviewer consensus protocol. Records where the intervention intended to modify a pre-existing molecular or histopathological state rather than purely screen for occult cancer were segregated specifically for the interception operational framework, ensuring that screening and diagnostic trials were strictly decoupled from biological interception studies.

### Eligibility criteria and study selection

To maximize transparency and address the lack of native registry tags for interception, all classifications were conducted independently by two reviewers, with discrepancies resolved through consensus arbitration involving a senior investigator. Records presenting overlapping characteristics (such as chemoprevention trials intersecting with early detection) were audited to ensure that the primary biological intent modifying a pre-existing state versus screening dictated the final categorization. Inter-reviewer agreement was not formally quantified using an indicator such as Cohen's kappa.

Eligible records were studies registered in ClinicalTrials.gov that addressed cancer prevention, risk-reduction, chemoprevention, premalignant or precursor condition, interception-related, early detection or minimal residual disease-related research question. Studies were excluded when the registry information was insufficient to determine their relevance to the research question or when they represented treatment of established cancer without a prevention, interception, or disease-control objective.

Because ClinicalTrials.gov lacks native tags for ‘cancer interception’ or ‘post-treatment disease control’, studies cataloged under broad fields such as ’Supportive Care’, ‘Basic Science’, or miscellaneous designations were re-evaluated against our operational framework. This harmonization ensured that trials targeting minimal residual disease or premalignant lesions were systematically classified according to their biological objective rather than the registry's administrative label, thereby mitigating the structural limitations of legacy database taxonomies.

### Operational framework for prevention and interception research

Because the terminology used in the literature is heterogeneous, the study population and research objective were considered when interpreting the prevention or interception context of the identified studies. For this study, cancer interception was defined as an intervention delivered after the identification of a measurable biological, molecular, histopathological, or clinically recognized state associated with an increased probability of progression to invasive cancer, with the primary objective of preventing or delaying progression to clinically manifest malignancy. Primary prevention refers to interventions in individuals without detectable premalignant disease who are at increased risk of cancer. Interception refers to interventions delivered during an early biological or premalignant state with the objective of preventing or delaying progression to invasive cancer. Early detection refers to strategies intended to identify clinically occult or early-stage disease to facilitate diagnosis or treatment before more advanced disease develops. Post-treatment disease-control studies include interventions aimed at reducing recurrence or controlling minimal residual disease after treatment of established cancer.

For example, studies targeting premalignant lesions or measurable molecular abnormalities with the objective of preventing progression to invasive cancer were classified as interception, whereas studies addressing recurrence or minimal residual disease after treatment of established cancer were classified as post-treatment disease control.

Given the inherent heterogeneity of the terminology used in oncology research, we established an operational framework to classify the identified studies. This framework maps our conceptual categories primary prevention, early detection, interception, and post-treatment control to the registry-defined study purposes. Because ClinicalTrials.gov lacks native tags for ‘cancer interception’ or ‘post-treatment disease control’, studies cataloged under broad fields such as ’Supportive Care’, ‘Basic Science’, or miscellaneous designations were re-evaluated against our operational framework. This harmonization ensured that trials targeting minimal residual disease or premalignant lesions were systematically classified according to their biological objective rather than the registry's administrative label, thereby mitigating the structural limitations of legacy database taxonomies.

To ensure methodological traceability, the operational categorization followed a standardized decision algorithm applied to each record's core metadata (Conditions, Interventions, and Study Purpose fields). Specifically, records were evaluated sequentially: (1) if the study population comprised healthy individuals at risk without evidence of lesions, it was classified as *Primary Prevention*; (2) if the objective focused on screening, early diagnosis, or identifying occult lesions in asymptomatic populations, it was classified as *Early Detection and Diagnosis* (3) if the intervention targeted a defined molecular abnormality, genetic predisposition, or histological premalignant state to halt progression, it was classified as *Cancer Interception* and if the trial addressed minimal residual disease, recurrence prevention, or post-treatment surveillance following established cancer therapy, it was classified as *Post-treatment Disease Control*.

The proportional mapping of study purpose and operational categories was restricted to the 157 interventional studies, for which the registry provided standardized ‘Primary Purpose’ metadata. The 52 observational studies were not included in this categorical breakdown because ClinicalTrials.gov does not provide an equivalent standardized primary purpose field for these records. Accordingly, the descriptive analyses of the full study population are presented for all 209 eligible records, whereas the operational categorization is based exclusively on the 157 interventional studies. Cancer interception was operationally defined only within this interventional subset, as this category requires an intervention intended to halt or delay the progression of a defined molecular abnormality, genetic predisposition, or premalignant state. Study-level details supporting the categorization of the interventional records are provided in Supplementary Table S1.

### Variables extracted

The following study characteristics were extracted: recruitment status; study type; study purpose; participant age eligibility; sex eligibility; estimated enrollment; cancer localization; funding category; study phase; allocation; design and masking.

Recruitment status included not yet recruiting, recruiting, active but not recruiting, completed, terminated, withdrawn, and unknown. Study purpose was based on the corresponding ClinicalTrials.gov registry field and included the categories available in the registry. For interventional studies, allocation was classified as randomized or non-randomized when applicable. Intervention models included parallel, single-group, crossover, factorial, and sequential designs. Masking was classified according to the registry field. Study phases were summarized according to the ClinicalTrials.gov classification. Funding information was obtained directly from the ‘Funded By’ (funder_type) field reported in ClinicalTrials.gov. This field classifies each registered study into one of the registry's own predefined categories (NIH, Other NIH, Other U.S. Federal agency, Industry, Network, and Other), and these native registry categories were used as provided, without additional recoding, merging, or manual sub-classification by the study team. Because ClinicalTrials.gov does not disclose the specific identity or institutional nature of funders classified under the broad ‘Other’ category, this label could not be further disaggregated using the available registry metadata. To enhance clarity and maintain standard oncological reporting, cancer-site categories extracted from the registry records were systematically harmonized and grouped into standardized anatomical and pathological classifications. Cancer localization was standardized into predefined anatomical or organ-system categories based on the primary cancer or condition reported in the ClinicalTrials.gov record. Studies involving multiple cancer sites were classified as Various. Specifically, specific organ subtypes with low frequencies (such as individual genetic or musculoskeletal records) were aggregated into broader categories, renal and urinary entries were consolidated into a unified *Genitourinary* category.

### Statistical analysis

Categorical variables were summarized as frequencies and percentages. Continuous variables were summarized using the median and interquartile range because enrollment distributions were expected to be skewed. Associations between categorical variables were assessed using the Fisher-Freeman-Halton exact test when contingency tables contained small or zero cell counts. Cramér's V was used as a measure of effect size for some contingency tables analyzed. Because conventional thresholds for interpreting Cramér's V vary according to table dimensionality (Cohen, 1988), the degrees of freedom (df = min(R−1, C−1)) of each contingency table were verified prior to interpretation. Tables where Cramér's V was analyzed included a dichotomous study-type variable (Interventional vs. Observational), yielding df = 1 regardless of the number of categories in the other variable; accordingly, Cohen's (1988) conventional thresholds for 1-degree-of-freedom tables (small ≈ 0.10–0.29, moderate ≈ 0.30–0.49, large ≈ over 0.50) were applied directly. (https://www.taylorfrancis.com/books/mono/10.4324/9780203771587/statistical-power-analysis-behavioral-sciences-jacob-cohen).

## Results

### Study selection

The search identified 296 records. After exclusion of 75 records that were not relevant to the cancer prevention or interception research question and 12 records with insufficient information for classification, 209 studies were included in the final analysis (Figure 1).

**Figure 1:**
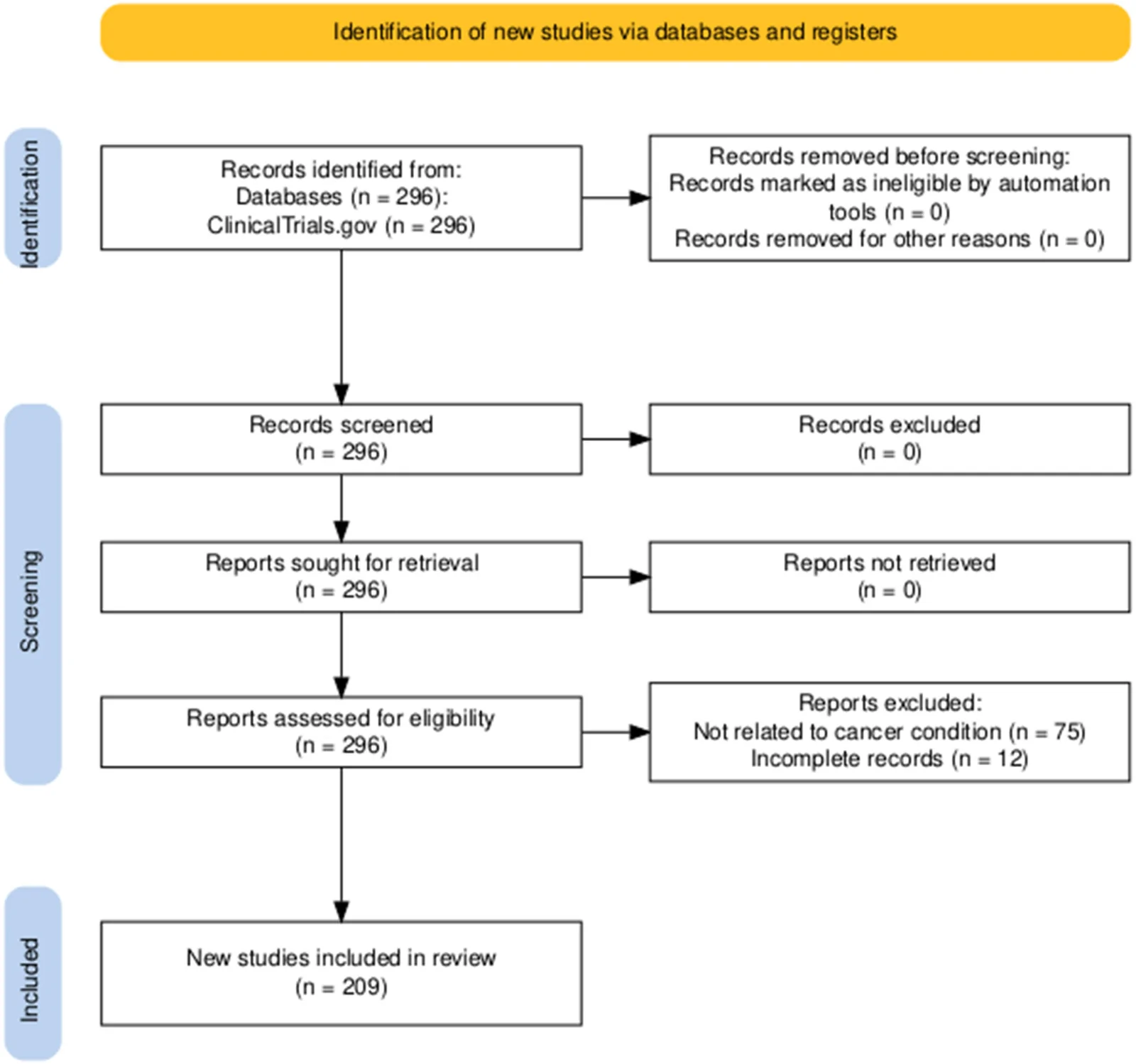
Flow diagram showing the identification, screening, and inclusion of studies assessing cancer prevention and interception research in ClinicalTrials.gov.

### Overall characteristics of included studies

Among the 209 included studies, 110 (52.6%) were completed, 41 (19.6%) were recruiting, and 18 (8.6%) had not yet started recruitment. Nineteen studies (9.1%) had an unknown recruitment status, while the remaining recruitment categories were infrequent. Regarding eligibility criteria, 149 studies (71.3%) included all sexes, 54 (25.8%) were restricted to females, and 6 (2.9%) to males. With respect to age eligibility, 153 studies (73.2%) included both adults and older adults, while the remaining studies included adults, older adults, and/or children in different combinations. These age and sex distributions represent registry-specified eligibility criteria rather than the demographic characteristics of enrolled participants. Funding was classified as ‘Other’ in 183 studies (87.6%), followed by industry funding in 14 (6.7%). NIH and other government funding were each reported for 5 studies (2.4%), whereas federal and network funding were each reported for 1 study (0.5%). The predominance of the ‘Other’ category limited further characterization of funding sources based on the registry classification alone. Most studies were classified as ‘Not Applicable’, representing the predominant study-phase category in the registry. Among the 157 interventional studies, 135 (86.0%) were classified as having no applicable study phase. Among studies assigned a specific phase, Phase 2 was the most frequently reported (4.6%), followed by Phase 3, Early Phase 1 and Phase 4. Phase 1 and combined Phase 1/Phase 2 studies were each reported in 1 study (0.6%). Overall, conventional phase classifications were uncommon, with most interventional studies classified as ‘Not applicable’. This distribution reflects the phase classifications recorded in ClinicalTrials.gov and includes studies for which the registry designated the study phase as ‘not applicable’. Estimated enrollment varied substantially across studies, with a median of 150 (IQR 61.5–533.5) (Table 1).

**Table 1:** Characteristics of included studies.

Status	No (%)
Recruitment	
Active not recruiting	10 (4.8%)
Completed	110 (52.6%)
Enrolling by invitation	3 (1.4%)
Not yet recruiting	18 (8.6%)
Recruiting	41 (19.6%)
Suspended	2 (1.0%)
Terminated	3 (1.4%)
Unknown	19 (9.1%)
Withdrawn	3 (1.4%)
Total	209 (100%)
Gender	
All	149 (71.3%)
Female	54 (25.8%)
Male	6 (2.9%)
Total	209 (100%)
Age	
Adult	19 (9.1%)
Adult, older adult	153 (73.2%)
Child	8 (3.8%)
Child, adult	11 (5.3%)
Child, adult, older adult	16 (7.6%)
Older adult	2 (1.0%)
Total	209 (100%)
Funding	
Federal	1 (0.5%)
Industry	14 (6.7%)
Network	1 (0.5%)
NIH	5 (2.4%)
Other	183 (87.6%)
Other gov	5 (2.4%)
Total	209 (100.0%)
Phases	
Early phase 1	4 (2.5)
Not applicable	135 (86.0)
Phase1	1 (0.6)
Phase1|phase 2	1 (0.6)
Phase 2	7 (4.5)
Phase 3	6 (3.8)
Phase 4	3 (1.9)
Total	157^a^ (100.0%)
Estimated enrollment	Median (IQR) | 150 (61.5–533.5) |

a Interventional trials.

### Study design and phase

Among interventional studies, randomized allocation was more common than non-randomized allocation. Parallel-group designs predominated, followed by single-group designs, while crossover, factorial, and sequential designs were uncommon. Most interventional studies were unmasked, with single and double-masked designs representing smaller proportions (Table 2).

**Table 2:** Allocation, design, and masking characteristics of interventional studies.

Variable	Category	No (%)
Allocation	Non_randomized	52 (33.1%)
	Randomized	105 (66.9%)
Design	Crossover	2 (1.3%)
	Factorial	4 (2.5%)
	Parallel	102 (65%)
	Sequential	3 (1.9%)
	Single group	46 (29.3%)
Masking	None	102 (65%)
	Single	28 (17.8)
	Double	20 (12.7%)
	Triple	2 (1.3%)
	Quadruple	5 (3.2%)
Total		157 (100%)

### Cancer localization

The distribution of interventional versus observational study designs across standardized cancer localizations was evaluated. A Fisher-Freeman-Halton exact test revealed no statistically significant association between harmonized cancer localization and study type (*p* = 0.09) (Table 3). Cramér's V was 0.32 (df = 1), indicating a moderate effect size.

**Table 3:** Distribution of studies by localization of malignancy and study type.

System	Interventional	Observational	Total	*p* value^a^
Dermatology	11 (5.3%)	3 (1.4%)	14 (6.7%)	
Endocrine	1 (0.5%)	3 (1.4%)	4 (1.9%)	
Gastrointestinal	35 (16.7%)	14 (6.7%)	49 (23.4%)	
Genetic	1 (0.5%)	0 (0.0%)	1 (0.5%)	
Gynecological	45 (21.5%)	8 (3.8%)	53 (25.4%)	
Head and Neck	3 (1.4%)	4 (1.9%)	7 (3.3%)	
Hematological	13 (6.2%)	1 (0.5%)	14 (6.7%)	
Musculoskeletal system	1 (0.5%)	0 (0.0%)	1 (0.5%)	0.09
Neuroendocrine	2 (1.0%)	0 (0.0%)	2 (1.0%)	
Neurological	1 (0.5%)	1 (0.5%)	2 (1.0%)	
Other	17 (8.1%)	7 (3.4%)	24 (11.5%)	
Genitourinary	5 (2.4%)	2 (1.0%)	7 (3.3%)	
Respiratory	9 (4.3%)	7 (3.3%)	16 (7.7%)	
Various	13 (6.2%)	2 (1.0%)	15 (7.2%)	
Total	157 (75.1%)	52 (24.9%)	209 (100.0%)	

a Fisher-Freeman-Halton exact test, *p* = 0.09 (global association between study type and localization).

### Age and sex eligibility characteristics of included studies

Among the 209 included studies, 153 (73.2%) specified eligibility for both adults and older adults, including 116 (55.5%) that allowed all sexes, 34 (16.3%) restricted eligibility to females, and 3 (1.4%) to males. Nineteen studies (9.1%) included adults only, with 7 (3.4%) allowing all sexes, 9 (4.3%) restricted to females, and 3 (1.4%) to males. Eight studies (3.8%) included children only, all of which allowed eligibility for all sexes. Eleven studies (5.3%) included both children and adults, while 16 (7.7%) included children, adults, and older adults. Among these studies, eligibility for all sexes was specified in 6 (2.9%) and 11 (5.3%), respectively. Only 2 studies (1.0%) were restricted to older adults, with 1 (0.5%) allowing all sexes and 1 (0.5%) restricted to females. Overall, 149 studies (71.3%) included all sexes, 54 (25.8%) were restricted to females, and 6 (2.9%) to males (Table 4).

**Table 4:** Distribution of studies by age eligibility and sex eligibility.

Age group	All	Female	Male	Total
Adult	7 (3.4%)	9 (4.3%)	3 (1.4%)	19 (9.1%)
Adult, older adult	116 (55.5%)	34 (16.3%)	3 (1.4%)	153 (73.2%)
Child	8 (3.8%)	0 (0.0%)	0 (0.0%)	8 (3.8%)
Child, adult	6 (2.9%)	5 (2.4%)	0 (0.0%)	11 (5.3%)
Child, adult, older adult	11 (5.3%)	5 (2.4%)	0 (0.0%)	16 (7.7%)
Older adult	1 (0.5%)	1 (0.5%)	0 (0.0%)	2 (1.0%)
Total	149 (71.3%)	54 (25.9%)	6 (2.9%)	209 (100.0%)

### Funding and study type

The ‘Other’ funding category was the most frequent classification, indicating that the registry-level category encompasses heterogeneous funding sources that could not be further differentiated using the available registry information. A statistically significant association was observed between funding category and study type (*p* = 0.028) (Table 5). Cramér's V was 0.25 (df = 1), corresponding to a small magnitude under the df = 1 interpretation framework. However, this association should be interpreted cautiously because most studies were classified under the heterogeneous ‘Other’ category.

**Table 5:** Distribution of studies by funder type and study type.

Type of Funder	Interventional	Observational	Total	*p* value^a^
Federal	0 (0.0%)	1 (0.5%)	1 (0.5%)	
Industry	11 (5.3%)	3 (1.4%)	14 (6.7%)	
Network	1 (0.5%)	0 (0.0%)	1 (0.5%)	
NIH	1 (0.5%)	4 (1.9%)	5 (2.4%)	0.028
Other	141 (67.5%)	42 (20.1%)	183 (87.6%)	
Other_gov	3 (1.4%)	2 (1.0%)	5 (2.4%)	
Total	157 (75.1%)	52 (24.9%)	209 (100.0%)	

a Fisher-Freeman-Halton exact test, *p* = 0.028 (global association between study type and funder type; the *p*-value is shown once for the overall table, not specific to the NIH row).

### Study purpose categories

The alignment between our conceptual framework and the study-purpose categories reported in ClinicalTrials.gov reveals significant heterogeneity in how prevention-oriented research is classified. From interventional studies, research focused on early detection and diagnosis (category 2) comprised 54 (34.4%) studies, mapped to the registry's ’Screening’ and ‘Diagnostic’ fields. However, research addressing cancer interception and post-treatment disease control (category 3), which accounted for 52 studies (33.1%), lacked a distinct registry designation. Primary prevention and chemoprevention were represented by 51(32.5%) studies aligning with the registry's ‘Prevention’ category (category 1). Consequently, these studies were dispersed across broader, non-specific fields such as ’Supportive Care’, ‘Basic Science’, and ‘Health Services Research’. These categories reflect the operational classification applied to interventional studies (*n* = 157) and should not be interpreted as a categorization of all 209 eligible records. This distribution underscores the limitation of relying solely on registry-defined metadata to characterize the evolving cancer interception landscape (Figure 2).

**Figure 2:**
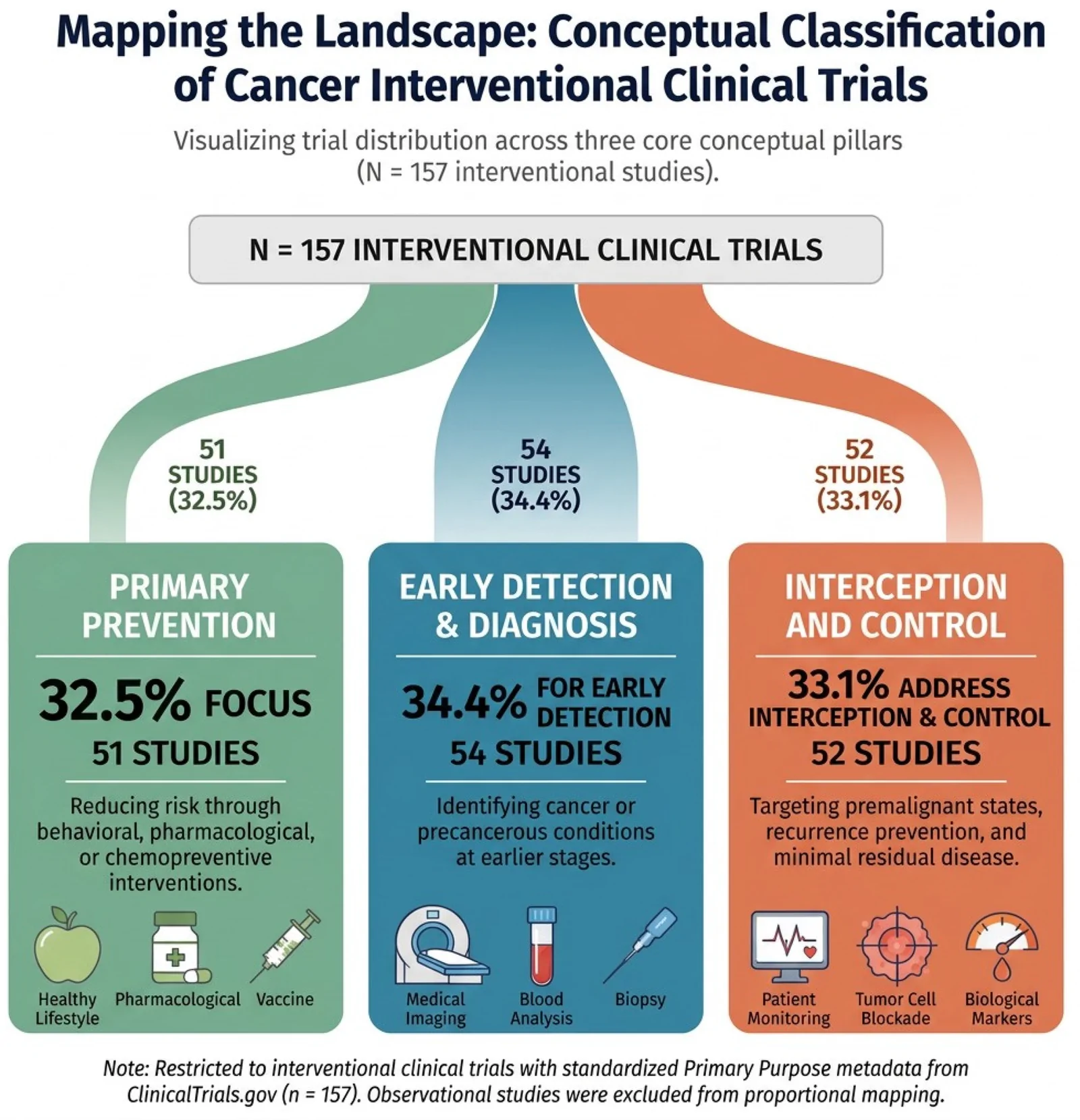
Mapping between operational conceptual categories and ClinicalTrials.gov study purpose categories (*N* = 157 interventional studies). Proportional distribution of interventional clinical trials (*n* = 157) demonstrating the mapping between the study's operational framework and registry-mandated fields. Categories include Primary Prevention (*n* = 51, 32.5%), Early Detection & Diagnosis (*n* = 54, 34.4%), and Cancer Interception and Post-treatment Disease Control (*n* = 52, 33.1%).

### Structural limitations of the registry

These structural limitations are accompanied by proposed operational solutions, which are further developed in the policy recommendations below. The first major limitation concerns the study phase mismatch, evidenced by a high proportion of entries classified as ‘Not Applicable’. To resolve this, the table proposes introducing a dedicated ‘Prevention/Interception Stage’ classification encompassing options like primary prevention, biological interception, or post-treatment control. Additionally, heterogeneous funding poses a significant challenge, with 87.6% of studies grouped under a broad ‘Other’ category that obscures true sponsor distributions (Table 6). The suggested solution is to mandate the disaggregation of funding sources to explicitly distinguish among philanthropic, academic institutional, cooperative group, and industry sponsors. The framework also highlights an ambiguous target population, noting a persistent lack of distinction between healthy risk cohorts and pre-invasive or precursor states. Adding a structured metadata field for ‘Baseline Biological/Risk Status’ such as genetic risk, molecular abnormality, or histological premalignant lesion is recommended to address this gap.

**Table 6:** Current registry limitations in cancer prevention/interception trials and proposed operational solutions.

Current registry limitation	Identified reporting gap	Proposed operational solution/new subfield
Study phase mismatch	High proportion of ‘Not Applicable'phase entries.	Introduce a ‘Prevention/Interception Stage’ classification (e.g., Primary, Biological Interception, Post-treatment).
Heterogeneous funding	87.6% classified under broad ‘Other’ category.	Mandatory disaggregation of funding source (Philanthropic, Academic Institutional, Cooperative Group, Industry).
Ambiguous target population	Lack of distinction between healthy risk cohorts and pre-invasive/precursor states.	Add a structured metadata field for ‘Baseline Biological/Risk Status’ (e.g., Genetic Risk, Molecular Abnormality, Histological Premalignant Lesion).
Intervention intent	Overlap between screening, chemoprevention, and interception in search terms.	Implement standardized tagging for ‘Primary Trial Objective’ to separate early detection from progression interruption.

Finally, overlapping intervention intent between screening, chemoprevention, and interception complicates search strategies, prompting the recommendation to implement standardized tagging for the ‘Primary Trial Objective’ to cleanly separate early detection from disease progression interruption.

## Discussion

### Principal findings

This study provides a registry-based characterization of cancer prevention and interception-related research registered in ClinicalTrials.gov. The principal findings were the predominance of interventional studies, the concentration of research in gynecological and gastrointestinal malignancies, the emphasis on adult and older adult populations, the predominance of randomized parallel-group designs among interventional studies, and the substantial heterogeneity of funding and registry classifications.

ClinicalTrials.gov has registered over 345,000 interventional and observational studies. Managed by the National Library of Medicine, it provides an accessible platform for healthcare professionals, researchers, and the public. Analysis of trial metadata reveals trends in study design, sample size, composition, and funding over time (13).

Methodological patterns also reveal distinct operational challenges: whereas conventional therapeutic oncology trials fit neatly into standard trial phases and recognized registry categories, our dataset demonstrates that prevention and interception research frequently operates outside these conventional pipelines, often leading to unclassified study phases and broader operational definitions (14). The most important implication is that cancer interception does not yet appear to constitute a clearly bounded research category within the registry. Studies identified through prevention and interception-related terminology may encompass primary prevention, early detection and diagnosis, and cancer interception and post-treatment disease control, including specific activities such as chemoprevention, management of premalignant states, risk reduction, and recurrence prevention. These activities may be biologically and clinically related, but they involve different populations, endpoints, intervention thresholds, and ethical considerations.

Some studies across various medical specialties demonstrate a consistent trend towards randomized, parallel-group designs in clinical trials, consistent with our findings. The prevalence of open-label studies, especially in earlier phases of research, reflects practical considerations and the nature of interventions. Funding sources vary, with a notable proportion from ‘Other’ categories, aligning with our observations. These comparisons underscore the methodological similarities and differences across specialties, highlighting the diverse approaches to clinical trial design and reporting (15–18).

### The current registry structure does not clearly capture cancer interception

The heterogeneity observed in this analysis indicates that broad searches using prevention- and interception-related terminology may retrieve studies addressing prevention, screening, diagnosis, supportive care, and other research purposes. This finding highlights the importance of interpreting registry-defined categories in conjunction with study-level information when characterizing the cancer prevention and interception landscape.

The frequent use of broad or non-specific categories, including ‘Not Applicable’ for study phase and ‘Other’ for funding, further limits the ability to characterize the field using registry metadata alone.

This issue is particularly important because interception research often involves populations that are neither healthy individuals undergoing primary prevention nor patients with clinically established cancer. Individuals may instead have inherited risk, molecular abnormalities, precursor lesions, or other intermediate biological states. Registry fields that explicitly capture these states could improve the visibility and reproducibility of prevention-oriented clinical research.

### Distribution across cancer localizations

The concentration of studies in gynecological and gastrointestinal malignancies is not merely a reflection of disease burden; rather, it underscores a fundamental methodological disparity in oncology research. These cancer types are characterized by well-defined precursor states and identifiable biological biomarkers such as cervical intraepithelial neoplasia or colorectal adenomas that provide feasible endpoints for interception trials. In contrast, for malignancies where the earliest biological changes remain elusive or clinically occult, the barrier to designing reproducible interception studies remains high. This suggests that the current registry landscape is heavily biased toward ‘high-feasibility’ biological pathways, potentially leaving critical unmet clinical needs in less-defined interception domains poorly addressed. However, the distribution of studies should not be interpreted as a direct measure of disease burden or unmet clinical need. Registry representation is influenced by scientific feasibility, availability of biomarkers, research infrastructure, funding, and the likelihood that a prevention endpoint can be measured within a reasonable follow-up period.

### Trial design and methodological implications

The predominance of randomized and parallel-group designs among interventional studies is consistent with the need to generate causal evidence for preventive interventions. At the same time, the relatively frequent use of single-group and open-label designs may reflect the practical challenges of studying interventions in high-risk or premalignant populations, where recruitment can be difficult and blinding may be challenging depending on the intervention.

The interpretation of study phase also requires caution. Conventional phase classifications were developed primarily for therapeutic drug development and may not adequately represent prevention studies, behavioral interventions, devices, procedures, or studies evaluating progression of premalignant states. A prevention-specific classification framework may therefore improve the interpretability of registered studies.

### Funding and reporting transparency

The high frequency of ‘Not Applicable’ study phases and ‘Other’ funding categories reflects the structural challenges in adapting registries designed primarily for pharmacological therapeutic efficacy in invasive cancer to the context of precision prevention. A critical finding of our landscape analysis is that 87.6% of records were classified under the heterogeneous funding category ‘Other’. Because ClinicalTrials.gov groups institutional support, local academic funding, and diverse philanthropic grants under this unrefined label, current metadata cannot be reliably analyzed to determine true sponsor distributions. Specifically, the overwhelming proportion of records cataloged under the ‘Other’ label (87.6%) precludes unraveling the true financial support structure of preventive and interception oncology. Because the repository groups nonprofit philanthropic foundations, institutional academic budgets, internal university funds, and local research grants under this single generic term, the lack of standardized subclassification severely restricts our ability to infer which sectors drive this field. Without transparent disaggregation in the original registry metadata, it remains impossible to determine whether molecular interception trials rely primarily on philanthropic capital or institutional academic allocations, which in turn limits direct comparability with funding patterns of conventional therapeutic trials.

Concretely, the available registry metadata do not permit us to determine the proportion of ‘Other’ classified studies that were academically, philanthropically, or institutionally funded; to compare funding patterns across primary prevention, interception, and post-treatment disease-control studies; or to assess whether funding source is associated with trial design, phase, or completion. We did not perform a manual, study-by-study sub-classification of the “Other” category (e.g., by consulting individual study records, sponsor websites, or institutional affiliations) because ClinicalTrials.gov does not require or consistently disclose the underlying funder identity for records coded as ‘Other’, and any manual attribution would have relied on incomplete or inferential information not verifiable across all records, introducing a substantial risk of misclassification. We view this as an important avenue for future work, ideally undertaken as a dedicated, independently validated sub-study rather than a *post hoc* exploratory exercise within the present descriptive analysis.

To ensure full study-level traceability, individual-level classification of all 209 included records is provided in Supplementary Table S1, allowing readers to verify the mapping between each study and its assigned operational category and to independently assess the robustness of the framework as applied in practice. We also substantially expand and clarify the methodological description of our operational categorization framework within the text. Specifically, we detailed the step-by-step decision algorithm and dual-reviewer consensus protocol used to assign each record based on its core metadata (condition, intervention description, and biological intent). This approach provides a transparent, reproducible framework allowing readers to understand precisely how studies were mapped across the prevention continuum without the necessity of an extensive cataloging appendix.

Consequently, the interpretation of the funding landscape in preventive oncology remains constrained by legacy metadata taxonomies that fail to capture the true diversity of non-NIH and non-industry financial ecosystems. Future registry enhancements should prioritize granular sponsor-type tracking to adequately reflect academic, institutional, and philanthropic investments in interception research.

Furthermore, conventional trial phase categories proved poorly suited to prevention research, as evidenced by the dominance of ‘Not Applicable’ entries. Traditional phase frameworks were built for therapeutic oncology and fail to capture biomarker modulation, interception of pre-invasive states, or long-term behavioral endpoints, limiting cross-trial comparability and registry usability. These broad, catch-all classifications, while serving standard clinical trial needs, appear to have limited utility for capturing the unique operational features of prevention and interception research. The reliance on these broad, catch-all classifications does not merely complicate data synthesis; it obscures the real-world visibility of prevention research. Future evolution of registry metadata to incorporate more granular fields, such as ‘premalignant disease state’ or ‘biomarker-driven eligibility’, could enhance the visibility and traceability of prevention-oriented research. Adopting such standards would likely facilitate more accurate quantification of global progress in cancer interception research within existing registry frameworks. More granular reporting of institutional, philanthropic, academic, nonprofit, and collaborative funding could improve understanding of how prevention-oriented research is supported and whether funding patterns differ between primary prevention, interception, and post-treatment disease control.

### Implications for future registries and research

Future clinical trial registries could improve the characterization of cancer interception research by incorporating structured fields for prevention stage, premalignant or precursor status, biomarker-defined risk, molecular disease status, interception intent, and the primary endpoint targeted by the intervention. Such fields could help distinguish prevention of cancer onset, prevention of progression, early detection, and prevention of recurrence.

Future studies should also examine temporal trends in registration, geographic distribution, biomarker use, intervention classes, and outcome definitions. Comparative analyses across multiple international registries could determine whether the patterns observed in ClinicalTrials.gov are generalizable to the global cancer prevention and interception research landscape.

When compared with broader landscape analyses of ClinicalTrials.gov such as general clinical trial tracking by Gresham and colleagues (14) our findings share structural similarities regarding the predominance of randomized parallel-group designs and adult populations in interventional research. However, notable discrepancies emerge when examining study phases and funding categories. While general oncology and therapeutic trials typically report high proportions of phase-specific drug development and transparent industry or federal sponsorship, our analysis of cancer prevention and interception reveals a heavy reliance on the ‘Other’ funding category and ‘Not Applicable’ phase classifications. These discrepancies stem from the inherent nature of prevention research: unlike therapeutic oncology trials that follow conventional drug-phase pipelines (Phase 1–3) for established tumors, prevention and interception studies frequently evaluate behavioral interventions, devices, nutritional components, or long-term biomarker progression in high-risk or premalignant cohorts. Consequently, the current standard registry taxonomies may not fully account for these distinct frameworks, contributing to the observed heterogeneity in reported study phases and funding categories.

Our findings call for a paradigm shift in how prevention-oriented research is reported. The prevailing lack of granularity in funding and study-intent reporting limits our ability to determine whether research is driven by academic, industrial, or collaborative non-profit initiatives. This knowledge gap is vital; without a transparent audit of funding patterns in cancer interception, policymakers cannot strategically target resources to bridge the ‘valley of death’ between preclinical prevention research and large-scale, registration-quality clinical trials.

Recent broad landscape analyses of ClinicalTrials.gov reveal critical operational benchmarks that contextualize our findings: while most registered trials enroll fewer than 100 participants and heavily favor early-phase drug interventions (up to 72.6%), completion rates within a five-year window hover around 60.5%. Furthermore, distinct sponsor-driven priorities shape the registry such as industry funding clustering predominantly around oncology and pharmacological interventions, contrasted with institutional prioritization of behavioral approaches alongside widespread challenges in data-sharing transparency, where over 70% of trials do not plan to share participant-level data (12).

When compared with the broader technological landscape of oncology trials, including emerging registry analyses of artificial intelligence applications in prostate cancer diagnostics, our findings reveal a contrasting pattern. While recent registry mappings demonstrate a growing concentration of trials evaluating diagnostic imaging, risk prediction, and decision-support tools, our analysis identified no dedicated ClinicalTrials.gov study-purpose category specifically representing cancer interception. Instead, studies addressing interception and post-treatment disease control were distributed across broader registry categories, including Supportive Care, Basic Science, and Health Services Research. This finding suggests that cancer interception is not yet adequately represented as a distinct clinical research category within standard registry metadata, potentially limiting its visibility and the reproducibility of registry-based landscape analyses. To address the structural and reporting deficiencies identified in our landscape analysis, we propose a set of targeted operational enhancements for clinical trial registries (summarized in Table 6). The high frequency of ‘Not Applicable’ phases and the overwhelming dominance of unrefined funding labels underscore the observed registry classifications suggest that current metadata may have limited granularity for consistently characterizing the prevention and interception continuum. Implementing standardized metadata subfields such as distinct ‘Prevention/Interception Stage’ tags, mandatory funding disaggregation, and structured descriptors for baseline biological or risk status would profoundly improve cross-trial comparability. These structural refinements are vital not only for enhancing data transparency and reducing reporting ambiguity, but also for enabling robust evidence synthesis and guiding future policy and funding allocations in preventive oncology.

To further refine these registry systems, we also propose the integration of standardized biomarker and risk-stratification tags (specifying genomic, molecular, or imaging-based criteria) alongside dedicated fields for longitudinal endpoint tracking (such as precursor lesion regression or time to invasive progression). Incorporating these specific metrics would resolve the current ambiguity between traditional screening and biological interception, ultimately providing researchers with a high-resolution framework to evaluate interventions targeting early carcinogenic states.

### Policy implications and practical recommendations

To transition our findings from descriptive critique into actionable policy, international registry administrators including the World Health Organization's International Clinical Trials Registry Platform (ICTRP) and ClinicalTrials.gov stewards must modernize metadata architectures to reflect the biological realities of modern oncology. Based on the structural limitations identified in our landscape analysis (summarized in Table 6), we put forward the following practical recommendations for clinical trial registries, policymakers, and clinical investigators:

1. Funding Disaggregation: Registry platforms should eliminate broad, catch-all funding categories (such as the 87.6% ‘Other’ entries observed in our dataset). Mandatory sub-categorization distinguishing philanthropic grants, academic institutional budgets, cooperative groups, and commercial sponsors is an essential prerequisite for economic transparency and accurate resource tracking.
2. Adoption of Prevention/Interception Stages: Traditional phase classifications designed for therapeutic drug development fail to capture long-term biological modulation or pre-invasive disease progression. Registries should incorporate dedicated tagging for ‘Prevention/Interception Stage’ (e.g., primary prevention, biological interception, post-treatment control).
3. Integration of Baseline Biological and Risk Status: To resolve the ambiguity between standard screening and biological interception, registries should introduce structured metadata fields capturing genomic, molecular, or histopathological risk markers (e.g., genetic predisposition, molecular abnormalities, or premalignant lesions).

For clinical investigators and public health stakeholders, based on this descriptive landscape, we suggest that standardized, prevention-specific reporting fields, such as explicit interception stages, biological risk profiling, and disaggregated funding metadata, could help registry authorities and clinical investigators improve the visibility, traceability, and cross-trial comparability of preventive oncology research. Whether adopting such fields would in practice optimize resource distribution or accelerate the clinical translation of cancer interception cannot be established from the present cross-sectional, registry-based analysis and remains a hypothesis for future evaluation.

### Strengths

This study provides a rigorous, systematic classification of a dataset (*n* = 209) of clinical trials, effectively bridging the gap between traditional oncological trial designs and modern cancer prevention and interception paradigms. By explicitly differentiating three operational categories—primary prevention, early detection and diagnosis, cancer interception and post-treatment disease control, the analysis establishes a clear and reproducible framework for future researchers. Furthermore, going beyond a purely descriptive critique, the investigation delivers concrete, structured proposals for clinical trial registries, such as ClinicalTrials.gov, including mandatory metadata subfields that directly address current reporting gaps in study phases, funding sources, and target populations.

### Limitations

Several methodological limitations must be acknowledged. First, our analysis was restricted to ClinicalTrials.gov, which may introduce geographic and structural bias toward U.S. centric research networks, federally funded initiatives, and trials registered within North American jurisdictions. Consequently, trials registered exclusively in major international registries, such as the European Union Clinical Trials Information System (CTIS), the Japan Registry of Clinical Trials (jRCT), or registries within the World Health Organization International Clinical Trials Registry Platform (ICTRP) network, were not captured. This limits the global generalizability of the landscape described here.

Second, registry-based research is inherently constrained by database-level limitations, including incomplete records, delayed status updates, inconsistent researcher classifications, and potential misalignment between standardized registry fields and the complex objectives of contemporary cancer prevention and interception trials.

Third, the predominance of records classified under the broad ‘Other’ funding category (87.6%) represents an important limitation for interpreting the funding landscape. ClinicalTrials.gov groups heterogeneous sources, including academic institutional budgets, local non-profit grants, and philanthropic support, within this administrative category, preventing a more granular assessment of sponsor distribution and financial support. Accordingly, these data should not be interpreted as reflecting the true balance of academic, philanthropic, industry, or governmental investment in cancer prevention and interception research, nor as evidence of causal relationships between funding source and trial characteristics. We did not manually reclassify the ‘Other’ category because this would have required inferring funder identity from information not systematically available or verifiable across all records, potentially introducing substantial misclassification. The lack of an exploratory sub-analysis or granular information in ClinicalTrials.gov regarding the legal nature of these sponsors not only prevents quantifying the actual contribution of academia versus philanthropy with precision but also restricts our capacity to contrast whether significant differences exist in trial design, phase, or methodological rigor according to their funding source. Consequently, the observed associations between study type and sponsorship must be interpreted with caution, reflecting a structural limitation of the registry rather than a defined investment pattern across primary prevention, interception, and post-treatment disease control.

Finally, although independent review and standardized criteria were used, the manual mapping of heterogeneous study purposes inevitably involved some degree of subjective interpretation. Future studies should therefore incorporate multiple international trial registries and, where feasible, more detailed verification of study purposes and funding sources to improve the comprehensiveness and validity and robustness of these descriptive findings.

## Conclusions

Cancer prevention and interception-related studies registered in ClinicalTrials.gov represent a heterogeneous research landscape encompassing risk reduction, chemoprevention, premalignant disease, early biological intervention, and post-treatment disease control. Our findings indicate that current registry taxonomies do not adequately capture these distinct clinical objectives, limiting the visibility and comparability of prevention and interception research. Standardized prevention-specific reporting fields, including explicit interception stages, biological risk profiles, and more detailed funding metadata, could improve the consistency and interpretability of registry data. However, whether such changes would optimize resource allocation or accelerate the clinical translation of cancer interception cannot be established from this cross-sectional registry-based analysis and remains to be evaluated in future research.
